# Fast, Spectroscopy-Based Prediction of In Vitro Dissolution Profile of Extended Release Tablets Using Artificial Neural Networks

**DOI:** 10.3390/pharmaceutics11080400

**Published:** 2019-08-09

**Authors:** Dorián László Galata, Attila Farkas, Zsófia Könyves, Lilla Alexandra Mészáros, Edina Szabó, István Csontos, Andrea Pálos, György Marosi, Zsombor Kristóf Nagy, Brigitta Nagy

**Affiliations:** 1 Department of Organic Chemistry and Technology, Budapest University of Technology and Economics, Műegyetem rakpart 3, H-1111 Budapest, Hungary; 2 Directorate General for Medicine Authorization and Methodology, Strategy, Development and Methodology Division, National Institute of Pharmacy and Nutrition, Zrínyi u. 3, H-1051 Budapest, Hungary

**Keywords:** dissolution prediction, artificial neural networks, extended release formulation, Raman spectroscopy, NIR spectroscopy, tablet compression

## Abstract

The pharmaceutical industry has never seen such a vast development in process analytical methods as in the last decade. The application of near-infrared (NIR) and Raman spectroscopy in monitoring production lines has also become widespread. This work aims to utilize the large amount of information collected by these methods by building an artificial neural network (ANN) model that can predict the dissolution profile of the scanned tablets. An extended release formulation containing drotaverine (DR) as a model drug was developed and tablets were produced with 37 different settings, with the variables being the DR content, the hydroxypropyl methylcellulose (HPMC) content and compression force. NIR and Raman spectra of the tablets were recorded in both the transmission and reflection method. The spectra were used to build a partial least squares prediction model for the DR and HPMC content. The ANN model used these predicted values, along with the measured compression force, as input data. It was found that models based on both NIR and Raman spectra were capable of predicting the dissolution profile of the test tablets within the acceptance limit of the f_2_ difference factor. The performance of these ANN models was compared to PLS models using the same data as input, and the prediction of the ANN models was found to be more accurate. The proposed method accomplishes the prediction of the dissolution profile of extended release tablets using either NIR or Raman spectra.

## 1. Introduction

Near-infrared (NIR) and Raman spectroscopy are constantly evolving techniques—their utilization in the pharmaceutical industry is spreading by the day. They are fast and non-destructive analytical methods which require no sample preparation [1,2]. Since they are based on different physical phenomena (Raman scattering and NIR absorption), these two methods are considered to be complementary, as Raman measurements are more sensitive to compounds with aromatic rings (such as most active pharmaceutical ingredients (APIs)), while NIR spectroscopy is better suited for samples with σ-bond systems (such as most tableting excipients) [1]. Although these techniques have been around for decades, their application in the pharmaceutical industry was facilitated by recent advances in their technology [3,4,5]. NIR spectrometers equipped with diode arrays, acousto-optic tunable filters or Fourier transform wavelength selectors enable much faster and more robust spectrum acquisition compared to grating instruments [6]. Raman spectroscopy required the development of strong, reliable laser sources and filters to separate the small amount of Raman photons from Rayleigh photons, which are present in numbers larger by several magnitudes [7]. Advances in fiber optics led to the development of Raman probes which allow on-line monitoring of processes [8]. These improvements were needed in order to overcome difficulties, such as fluorescence, poor sensitivity and reproducibility [8,9]. NIR and Raman spectroscopy have been applied for determining content uniformity [10,11], monitoring blending processes [12,13,14], for fluidized bed granulation and coating of tablets [15], continuous fluidized bed drying [16], identifying counterfeit drugs [17] and detecting polymorphs [18,19,20]. These analytical methods yield a large amount of data, as spectra generally consist of measurements at hundreds of wavelengths. In order to extract useful information from these spectra, various chemometric methods are required [21,22,23]. Among the most commonly used are principal component analysis (PCA) [24] and partial least squares (PLS) regression [25].

Dissolution testing has been a subject of scientific research since the pioneering work of Noyes and Whitney [26]. Although the pharmaceutical industry has applied it for a long time to confirm batch-to-batch consistency, it has received increased attention after the correlation between in vitro dissolution profiles and bioavailability was discovered [27,28,29,30]. In vitro dissolution profiles are often part of the target product quality profile of the modern quality by design approach [31], and are vital in the approval of new products or existing products after changes in technology [32]. However, this method is labor-intensive, destructive and the tablets measured represent only a negligible minority of the batch. A promising alternative to in vitro dissolution testing, recently recommended by the FDA, is the prediction of the dissolution profile based on spectroscopic data and chemometric models as a surrogate method [33]. In the 1990s and 2000s, several articles were published in which the authors used individual chemometric models for each time point of the dissolution curve [34,35]. More recently, Hernandez et al. developed a single PLS model to predict the percent drug released at 40 different time points for tablets which were compressed from powder mixtures subjected to different amounts of shear after mixing the components [36]. NIR spectroscopy was utilized by Pawar et al. to enable the real-time release testing of tablets made by continuous direct compression by prediction of the in vitro dissolution profile [32].

Artificial neural networks (ANNs) are computational methods which try to reproduce the information processing capabilities of the human brain [37]. A large variety of ANNs have been developed in the last few decades to solve different problems related to pattern recognition, classification, clustering, regression and optimizing [38]. An important advantage of ANNs is their ability to solve nonlinear problems using historical or incomplete data [37]. ANNs have also been proven to be useful tools for data fusion [39]. However, when applying ANNs, the risk of overfitting of the model should always be considered, which is a serious problem often associated with these systems. Another important issue is the enormous computing capacity required to calculate ANNs, which could make their utilization uneconomical [40]. However, the performance of computers has vastly developed, which has opened the way to their practical application. The pharmaceutical industry has also found its own way to utilize them. ANNs were employed to optimize formulations [41], to model in vitro–in vivo correlations [42], to build quantitative structure–activity relationship models [43] and to be utilized in pharmacological studies [44]. Machine learning was utilized in various cases to achieve a better understanding of the effect of critical process parameters on the formulation. Lou et al. used six different machine learning methods to model the effects of powder surface coating on the compactibility of the powder [45]. Various machine learning techniques were utilized by Millen et al. to predict the critical quality attributes of the product of a wet granulation process [46]. Han et al. used ANNs and deep neural networks (DNNs) to predict the disintegration time of oral disintegrating tablets [47]. ANNs are also able to predict in vitro dissolution profiles based on various input data. Ebube et al. used ANNs to predict the dissolution profile of tablets with three various compositions. They used the theoretical composition of the tablets as input variables [48]. Peh et al. applied an ANN to predict the dissolution profiles of theophylline pellets containing different ratios of microcrystalline cellulose and glyceryl monostearate. They used the amount of the two aforementioned materials, the time point of the measurement and the difference between the dissolution percentages of the two previous points as inputs. The f_2_ value was used to evaluate the similarity of the predicted and measured dissolution profiles [49]. These early works utilized the theoretical properties (e.g., theoretical composition) of the formulations as input data; therefore, their applicability is limited. The real-time monitoring of a manufacturing process requires a model which can predict dissolution profiles using in-line measured analytical data. Therefore, our aim was to create a model which uses the measured properties of the tablets as input.

Our created models purpose is to utilize the advances of spectroscopy and computer technology. New spectroscopic instruments are spreading in the pharmaceutical industry, and their application as a process analytical technology (PAT) has become common. The analytical data yielded by these instruments needs to be exploited to its full potential in order to extract as much information about the monitored system as possible. Our goal was to use the recorded spectra to measure the concentration of the drug and extended release matrix agent in the tablets. This information, along with the compression force of the tablets was used as input of an ANN model which predicts the in vitro dissolution profile of the tablets. The presented method is a faster alternative of the current dissolution testing, which obsoletes the inconvenient tasks associated with the current procedure (preparation of buffers, waiting several hours for the results, cumbersome cleaning), while it has the potential to evaluate a much larger portion of the produced tablets.

## 2. Materials and Methods

### 2.1. Materials

Drotaverine (DR) was obtained from Sigma-Aldrich (Munich, Germany). Hydroxypropyl methylcellulose (HPMC) K4M DC2 was a kind gift of Colorcon (Budapest, Hungary). Microcrystalline cellulose (MCC, Vivapur 200) was purchased from JRS Pharma (Rosenberg, Germany). Magnesium stearate (MgSt) obtained from Hungaropharma Ltd. (Budapest, Hungary) was used as a lubricant. Concentrated hydrochloric acid solution was purchased from Merck Ltd. (Darmstadt, Germany).

### 2.2. Methods

#### 2.2.1. Experimental Design

Tablets were made with a total of 37 different settings, from which four tablets per setting were selected for analysis. Of the settings, 27 originated from a 3^3^ factorial experimental design. This experimental design had the following factors: DR content (6%, 8%, 10%), HPMC content (10%, 20%, 30%) and compression force (63.8 MPa, 95.7 MPa, 127.6 MPa). In the additional 10 settings, two factors were set to the center point level, while the third was set to various levels inside and outside the predetermined set points. The experimental conditions for each setting are shown in Table 1. Thirty settings were used for the calibration of PLS models and as a training set for the ANN, while seven settings (1, 4, 14, 17, 27, 29, 34) were chosen for testing.

#### 2.2.2. Tablet Manufacturing on a Single Punch Tablet Press

All tablets consisted of four components: DR as the API (6–10 *w*/*w* %), HPMC as the sustained release agent (5–35 *w*/*w* %), MgSt as lubricant (1 *w*/*w* %) and MCC as filler and binder (54–88 *w*/*w* %). The API was blended with the HPMC and MCC excipients by manual mixing (shaking with regular changes in the direction) in a bottle for 5 min, and a mixture of 10 g was prepared in all cases. Then, 1% of MgSt was added to the blend and the mixture was shaken for an additional 2 min. On a Dott Bonapace CPR-6 single punch tablet press (Limbiate, Italy) equipped with 14 mm concave punches, the 500 mg tablets were compressed. The compression forces varied between 31.9 MPa and 159.5 MPa, as defined in the experimental design.

#### 2.2.3. Raman Spectroscopy

Raman spectroscopy measurements were carried out using a Kaiser Raman RXN2 Hybrid Analyzer (Ann Arbor, MI, USA) equipped by a Pharmaceutical Area Testing (PhAT) probe. A 785 nm diode laser source was used, and performance was set to 400 mW. Spectra were recorded in the range of 200–1890 cm^−1^ with 4 cm^−1^ spectral resolution. Reflection measurements were taken from a 6 mm diameter area of the tablets. Working distance was 25 cm in both cases. Two spectra were recorded for each tablet. Reflection and transmission spectra were measured for 5 and 30 s, respectively, and two scans were acquired in both cases.

#### 2.2.4. Fourier Transformation Near-Infrared Spectroscopy

NIR spectra were collected with a Bruker Optics MPA (Multi Purpose Analyzer) FT-NIR spectrometer (Billerica, MA, USA). A high intensity tungsten NIR source was used. NIR transmission spectra were collected in the 4000–15,000 cm^−1^ wavenumber range with 32 cm^−1^ spectral resolution. This method used the external transmission unit with an InGaAs detector. Background and sample spectra were collected by averaging 64 scans. The spectral range chosen for reflection NIR spectra was 4000–10,000 cm^−1^ with a resolution of 8 cm^−1^. Thirty-two scans were averaged for background and tablet spectra. An external fiber optic probe was used, and the detector was InGaAs. Tablets were placed under the probe so that the probe touched the surface of the tablets.

#### 2.2.5. In Vitro Dissolution Testing

The dissolution profiles of the tablets were recorded using a Hanson SR8-Plus dissolution tester (Chatsworth, CA, USA) following the United States Pharmacopoeia (USP) II method (paddle method). The dissolution medium was a 900 mL 0.1 N HCl solution at a temperature of 37 ± 0.5 °C. The rotational speed of the paddles was set to 100 rpm. The concentration of DR in the medium was measured with an on-line coupled Agilent 8453 UV-VIS spectrophotometer (Hewlett-Packard, Palo Alto, Santa Clara, CA, USA) using the absorbance of the medium at 356 nm, and 10 mm flow through cuvettes were used. The length of the dissolution run was 24 h. During this period, samples were taken at 53 time points (at 2, 5, 10, 15, 30, 45 and 60 min, after that once in every 30 min until 1440 min) using a Hanson Autoplus Maximizer 8 (Chatsworth, CA, USA) automatic syringe pump through 10 µm filters.

### 2.3. Data Analysis

#### 2.3.1. Experimental Design

The effect of experimental factors on the properties of the dissolution curve of the tablets was interpreted by using TIBCO Statistica 13 software (Palo Alto, CA, USA). The results of the first 27 settings were evaluated as a 3^3^ full factorial design. The independent variables (factors) were the nominal DR and HPMC content and the compression force of the tablets. Two models were calculated, and the dependent variables in these were the dissolution values measured at 15 and 960 min to represent both the initial and the later parts of the curve, respectively. The dissolution profiles were normalized to the mean (8%) DR content. The model fitted to the data used the linear and quadratic effect of the factors and the two-way interaction between the linear effects.

#### 2.3.2. Multivariate Data Analysis

The collected NIR and Raman spectra were analyzed using Matlab R2018a (MathWorks, Natick, MA, USA) and PLS Toolbox 8.6 (Eigenvector Research, Manson, WA, USA) software. PCA of the spectra was carried out in order to recognize patterns in spectral data depending on HPMC and DR content of the samples. Various pretreatments were tested on the four types of spectra, and PCA models were built in order to evaluate the effect of the experimental factors on the spectra. Similarly to PCA, PLS uses the linear combination of variables to create latent variables (LVs). However, LVs are calculated in a way that, apart from describing the variation of X data, also need to correlate with Y values [50]. The pretreated spectra were used to build PLS models to predict the DR and HPMC content of the test tablets; the methods resulting in the best models are described hereinafter. The contiguous block cross-validation method with 30 splits was used. Raman reflection and transmission spectra were baseline corrected (automatic Whittaker filter, *p* = 0.001, λ = 10,000), followed by standard normal variate and mean centering. NIR transmission spectra were derivated (1st derivate, Savitzky–Golay method, the number of points in the filter was five, and a second order polynomic function was fitted on the points) after which multiplicative signal correction and mean centering were applied, with NIR reflection spectra pretreated in the same way. The genetic algorithm (GA) was used as a variable selection method to enhance the predictive ability of the models. GAs were run with a population size of 64, a variable window width of 10 and a maximum LV number of 6. The best model was chosen from three replicate runs. The models were evaluated using the root mean square error of calibration (RMSEC), the root mean square error of cross-validation (RMSECV) and the root mean square error of prediction (RMSEP). The equations used to calculate these values are described by Porep et al. [2].

#### 2.3.3. Artificial Neural Network Models

ANN models were used to predict the dissolution profile of the tablets. ANNs were built by utilizing the Neural Network Toolbox included in Matlab R2018a (MathWorks, Natick, MA, USA). Feed-forward back-propagation networks were developed with the training functions Levenberg–Marquardt and Bayesian regularization. The mean-squared error was used as performance function. The networks consisted of three layers, the input layer had three neurons and the output layer had 53. For the training set, the inputs of the network were the theoretical DR and HPMC content of the tablets and the measured compression force. The training target was the measured dissolution profile of the training tablets, consisting of 53 time points. In the case of the test tablets, the inputs were the DR and HPMC content predicted by the PLS models and the measured compression force. The output of the built models was the predicted dissolution profile defined in 53 time points. The number of the neurons in the hidden layer was optimized by testing the network performance with different neuron numbers. Neuron number was gradually increased from 1 to 10—in each case the training step was repeated 100 times. In each training run, 15–15% of the training tablets were randomly chosen for cross-validation and testing. The resulting networks were evaluated by using them to predict the dissolution profile of the test tablets. The predicted and measured dissolution profile of the test tablets were compared by calculating the RMSEP values, and the models were characterized by the sum of these RMSEP values. For each neuron number, the average of the summed RMSEP was recorded, and the model with the lowest RMSEP value was chosen as the best model. In order to compare the performance of the spectroscopic methods, this process was repeated using three different inputs for the test tablets. In the first case, the DR and HPMC contents were predicted using NIR spectra, in the second, the Raman spectra were used, while in the third case, DR content was calculated from Raman and HPMC content from NIR spectra. The third input parameter was the measured compression force in all cases. The aforementioned steps were carried out using the Levenberg–Marquardt and Bayesian regularization training functions to find out which one was better suited for this purpose. The predictions of the best models were also compared by calculating the f_2_ similarity factor, which is a common way of comparing predicted and measured dissolution profiles [51]. This performs a logarithmic transformation of the squared vertical distances between the measured and the predicted values at each time point. An f_2_ value between 50 and 100 means that the two profiles can be accepted as equivalent. Models were evaluated by comparing the predicted and measured dissolution profiles based on the *f_2_* values (Equation (1)) [52].

(1) $$f_{2}=50log_{10}\left\{[1+\frac{1}{n}\sum_{t=1}^{n}w_{t}{\left(R_{t}-T_{t}{)}^{2}\right]}^{-0.5}\times 100\right\},$$

where *n* is the number of dissolution points, *R_t_* and *T_t_* are the measured and predicted dissolution values at time *t* and *w_t_* is an optional weighting factor.

In order to evaluate the possible advantage of ANNs, the predictions yielded by ANN models were compared to results obtained from a PLS model using the same inputs. These PLS models were built using the same inputs and targets as the ANN models. Input data was autoscaled (mean centered and divided by standard deviation), while target data was mean centered. The predictions of these PLS models were compared to the respective ANN models based on the average f_2_ value of all predicted profiles.

## 3. Results and Discussion

### 3.1. Evaluation of Experimental Design Results

**Figure 1:**
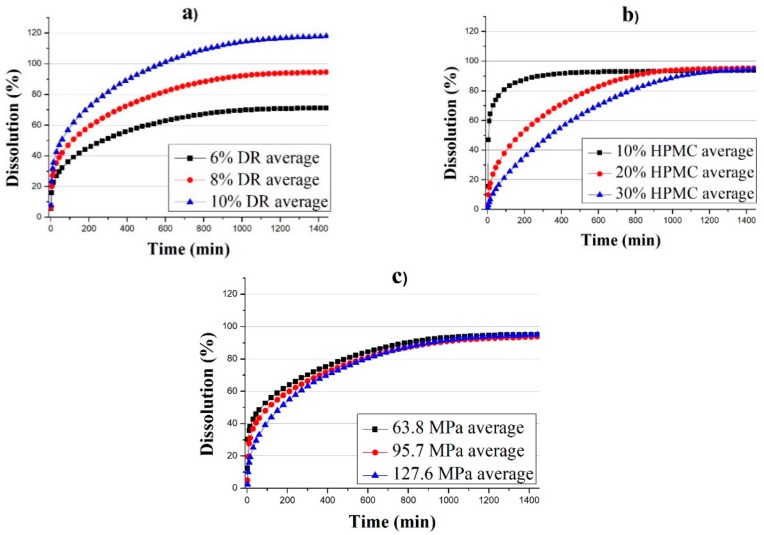
Average dissolution profiles sorted by (**a**) DR content; (**b**) HPMC content; (**c**) compression force.

The dissolution profiles of the tablets made according to settings 1–27 from Table 1 were used to evaluate the effect of the DR and HPMC content and the compression force. For a better visualization of the effects, dissolution profiles belonging to the same DR content, HPMC content and compression force were averaged (Figure 1). DR content determined the maximum dissolution achieved, but it did not seem to influence the shape of the curve. On the other hand, HPMC had a strong effect on the speed at which the drug was released. It is important to note the nonlinear effect of HPMC, as at 10%, the drug was released almost immediately, while at 20%, drug release became much slower. The difference was much smaller between the curves of 20% and 30% HPMC content. Compression force had only a slight effect on the initial part of the curve.

**Figure 2:**
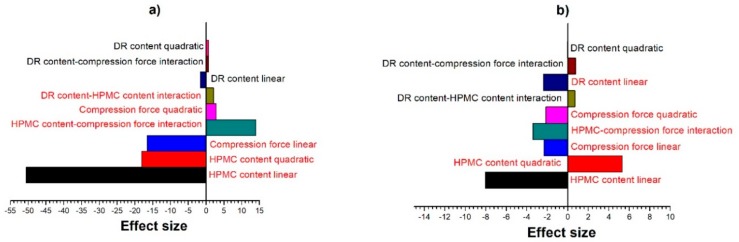
Effect of factors on the dissolution achieved at (**a**) 15 min and (**b**) 960 min. Effects were found significant at *p* = 0.05 are colored red. Interactions are linear.

The effect of the three parameters on the dissolution measured at 15 and 960 min was analyzed as a 3^3^ full factorial experimental design. The Pareto chart of the standardized effects on the dissolution at 15 min is displayed in Figure 2a. It is clear that HPMC content had the most significant effect, as its linear and quadratic terms had the two largest values. This result was not surprising, considering the fact that HPMC was added to the tablets to control the release of DR. When the concentration of HPMC is high enough, upon contact with water it forms a geling polymer network [53] and drug molecules reach the dissolution medium via diffusion through this layer. The compression force was also significant, as well as its interaction with the HPMC content. DR content had only a marginal effect at 15 min, that is, at the initial phase of the dissolution. The results were quite similar after 960 min (Figure 2b). HPMC level was still the dominating factor, but the relative importance of compression force decreased. At this point, the relative influence of DR content increased, and it became a significant factor with an effect similar to the compression force. The results of the experimental design show that a model that aims to predict the dissolution profile of these tablets needs to consider all three factors to determine the shape of the dissolution curve, while DR content was also vital in predicting the maximum dissolution achieved. It can be also concluded that for surrogate modeling of dissolution, all these factors need to be characterized accurately, for which tasking a single data source might not be sufficient.

### 3.2. Analysis of Spectroscopic Data

**Figure 3:**
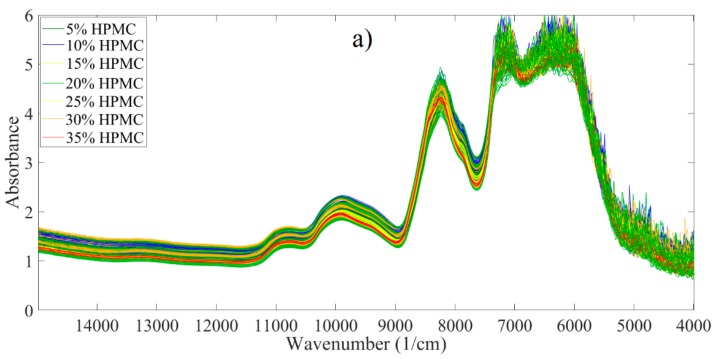

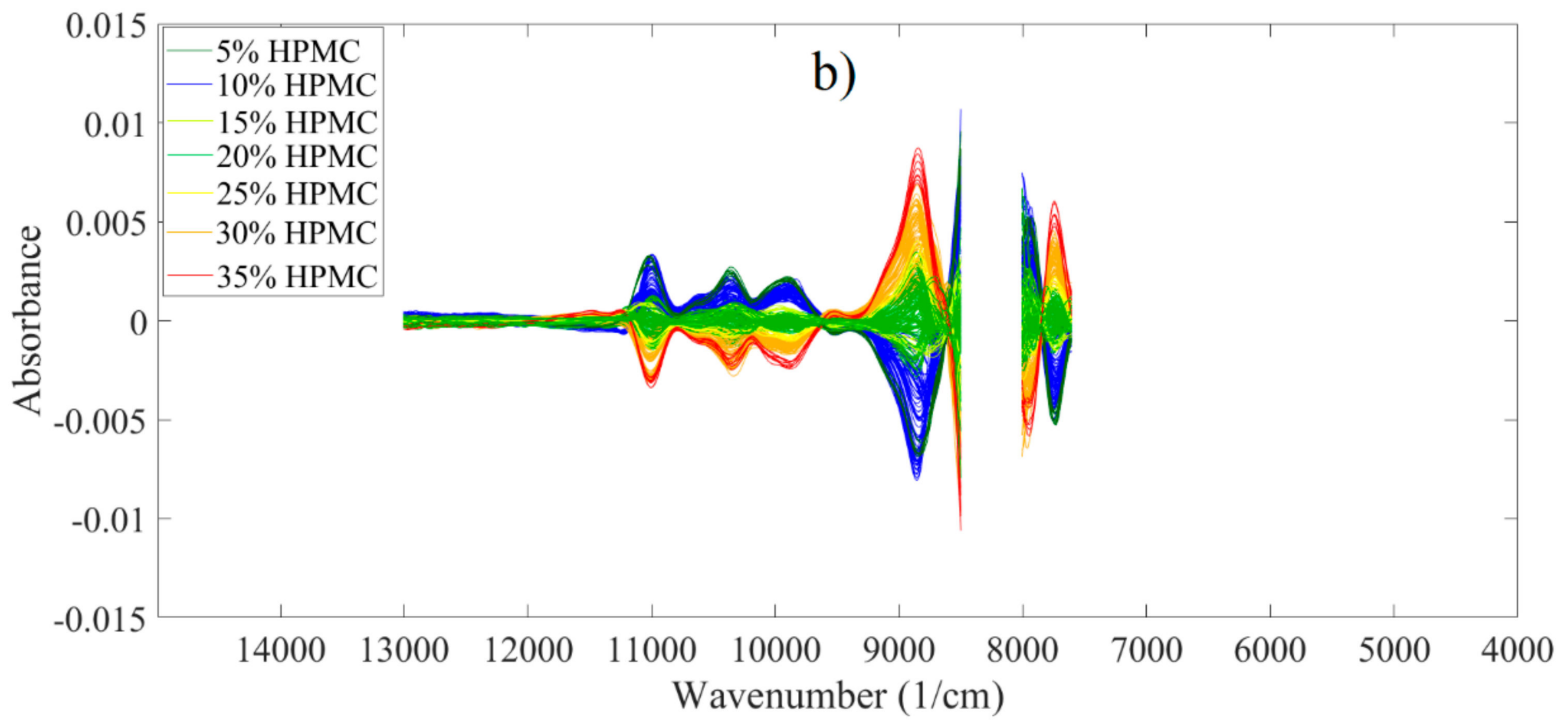
(**a**) Raw near-infrared NIR transmission spectra of training tablets; (**b**) preprocessed spectra.

The NIR and Raman spectra recorded in reflection and transmission mode were used to build PLS models to predict the DR and HPMC content of the test tablets. PCA was applied to the spectra, the methods which were found to be the most useful in extracting the required information from the data are described below. In the following section, only the analysis of transmission NIR and Raman spectra is described, as these were found to be more useful in predicting DR and HPMC content. PLS models based on reflection spectra yielded worse predictions in all cases. Figure 3a shows the raw NIR transmission spectra of the tablets.

The spectra provided noisy signals in the region below 7600 and at the 8000–8500 cm^−1^ band because of the high absorbance; therefore, these regions were excluded before further preprocessing steps. The first derivative of the spectra was calculated, as this process improves information extraction in cases when the signal of interest is contained in the sharper, narrower peaks of the spectrum. Multiplicative signal correction was applied in order to normalize the spectra and to remove the baseline offset, helping to remove differences between the spectra caused by multiplicative and additive effects during the collection of spectra. Mean centering was used before calculating the PCA model. The preprocessed spectra are shown in Figure 3b. After preprocessing, the samples with different HPMC contents could be clearly distinguished based on the different peak absorbance values. HPMC content correlated negatively with the intensity of peaks at 11,000, 10,300 and 9800 cm^−1^, and positively with the peaks at 8850 and 7750 cm^−1^.

**Figure 4:**
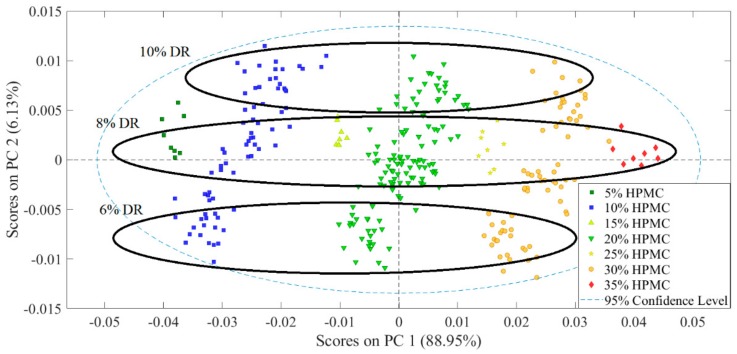
Score plot of preprocessed NIR transmission spectra.

The resulting PCA model used two principal components (PCs), explaining 88.95% and 6.13% of the total variance in the data, respectively. Observing the score plot of the first two PCs (Figure 4), the separation of the samples along the first PC could clearly be associated with the HPMC content, while the variance along the second PC could be identified as the DR content (see the groups along PC2 separated by DR content). See Figure S1 for the loading plots.

**Figure 5:**
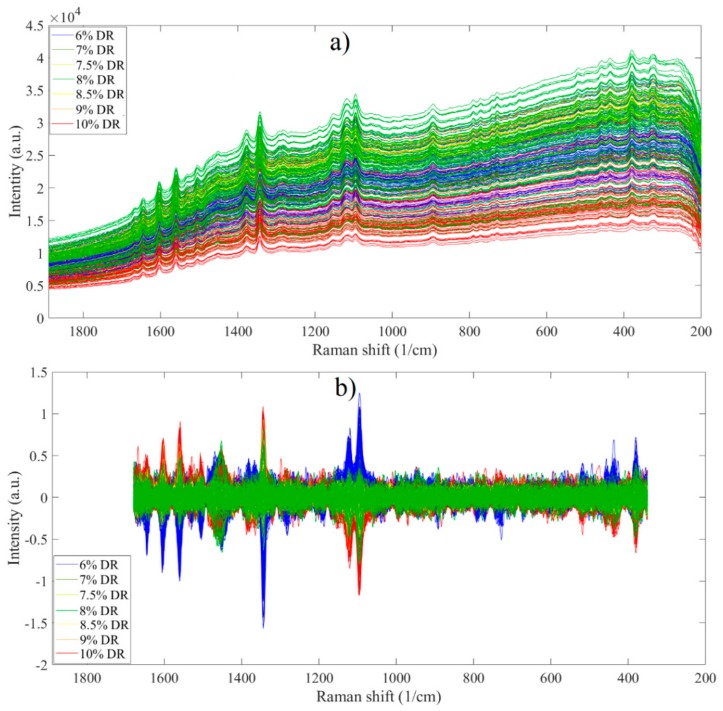
(**a**) Raw Raman transmission spectra of training tablets; (**b**) preprocessed spectra.

The raw Raman transmission spectra are presented in Figure 5a. The region above 1680 cm^−1^ was noisy and below 350 cm^−1^ a steep slope can be observed, which was caused by fluorescence. These regions were excluded before further examination of the data.

The baseline offset was removed by utilizing the automatic Whittaker filter method. Normalization of the spectra was carried out using standard normal variate. The spectra obtained this way are shown in Figure 5b. Mean centering was applied prior to calculating the PCA model. After preprocessing, the spectra consisted of several distinguishable, narrow peaks, and the intensity of these peaks correlated with the DR content. There was a positive correlation between DR content and the intensity of peaks at 1645, 1605, 1560 and 1345 cm^−1^, while DR content correlated negatively with the peak intensities at 1120 and 1090 cm^−1^, with these peaks being associated with HPMC and MCC.

**Figure 6:**
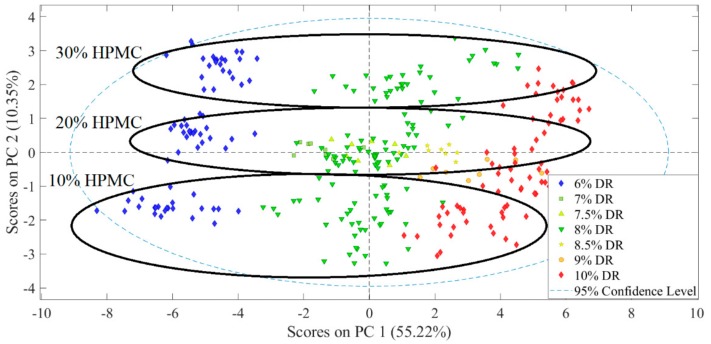
Score plot of preprocessed Raman transmission spectra.

A PCA model with two PCs was built based on these spectra. The first PC, responsible for 55.22% of the total variance, represented the DR content, while the second PC (10.35% variance explained) was associated with HPMC content. The score plot of these two PCs is shown in Figure 6. The samples with different DR content form distinguishable groups along the first PC, although the distance between the groups is smaller than in the case of HPMC content measured by NIR transmission. The loading plots are shown in Figure S2.

The reflection spectra were analyzed in a similar manner (data not shown). The pretreated spectra of the training set of tablets were used to build PLS models predicting the DR and HPMC content, which were applied to predict the content of the test tablets. The pretreated spectra may still contain regions which carry no useful information. Therefore, GA was used in order to choose the parts of the spectra which are the most valuable for the prediction. All four types of spectra were used to predict both DR and HPMC content of the test tablets, from which R^2^_p_ and RMSEP values were calculated. After the original models were built, GA was applied and it was evaluated as to whether it managed to improve the performance of the models. The parameters and the goodness indicators of the models predicting DR and HPMC content are shown in Table 2 and Table 3, respectively.

The PLS regression curves of models using transmission spectra are shown in Figures S3–S6. Based on the RMSE and R^2^ values, it was found that Raman transmission spectroscopy gave the best results for DR content, while for the prediction of HPMC content, NIR transmission had the best performance. Predictions of HPMC content had larger error values than predictions of DR content. This can be explained by the fact that in both NIR and Raman spectra, there were more peaks that can be associated with DR, and thus models trying to predict DR content have more information at their disposal. In the case of transmission spectra, GA was able to improve the performance of the models. Models based on reflection spectra yielded significantly worse results in all cases. NIR and Raman transmission spectra were used to predict both the DR and HPMC content of the test tablets. These predictions were used as input while testing the ANN models.

### 3.3. Predicting the Dissolution Profile by ANN

**Figure 7:**
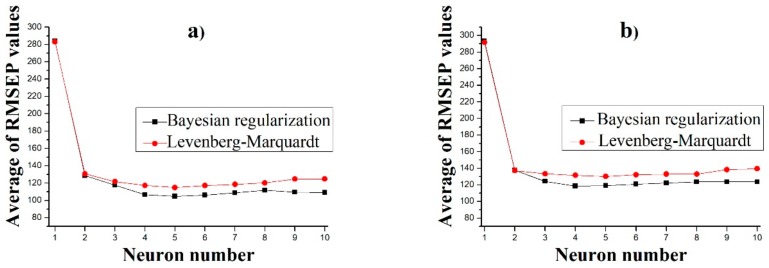

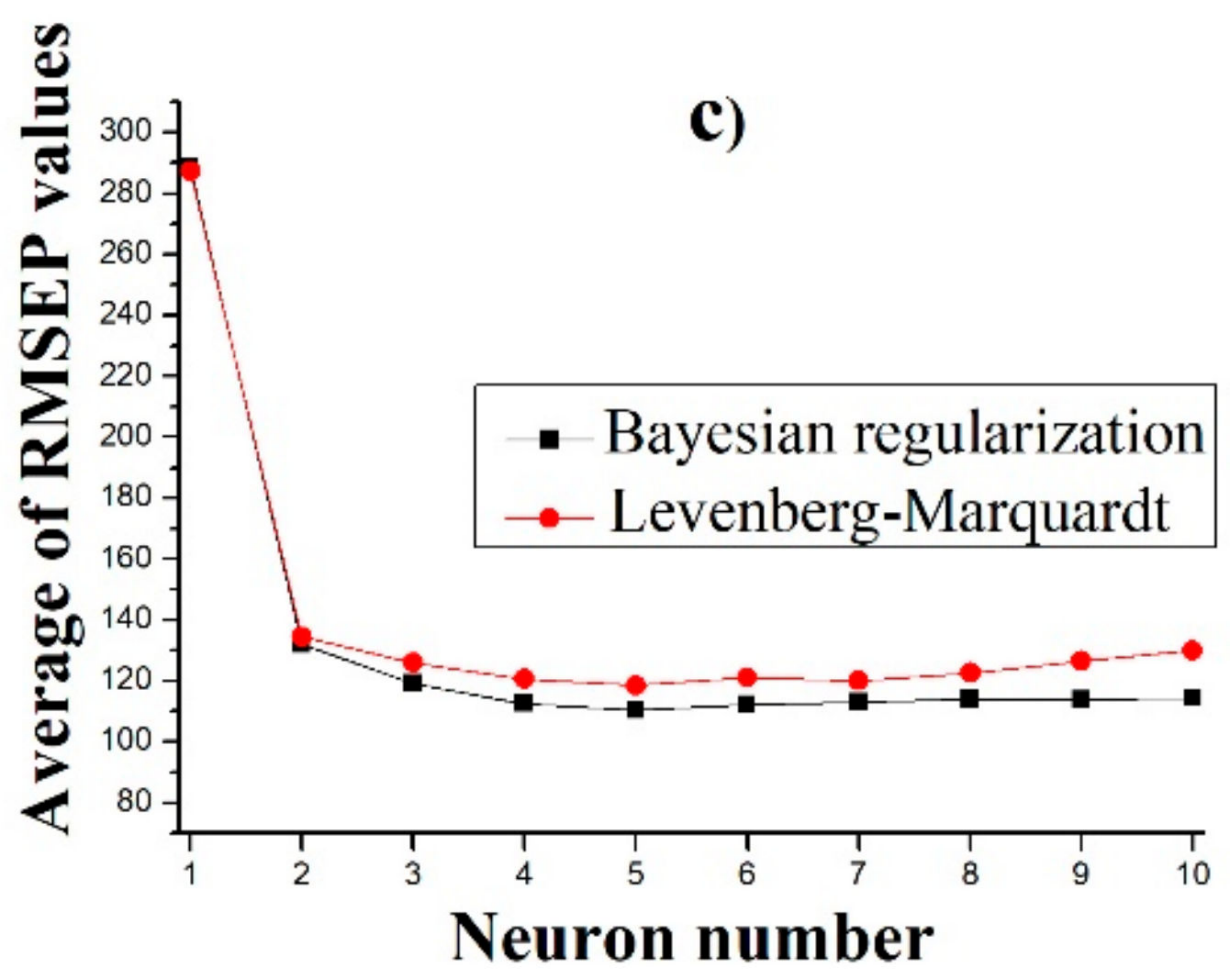
Average RMSEP values of predictions using DR and HPMC content predicted from (**a**) Raman, (**b**) NIR and (**c**) Raman and NIR spectra as input.

The average RMSEP values of the ANN models using three different validation input datasets are shown in Figure 7. The results show that one neuron yielded poor performance, yet the addition of a second neuron greatly improved the models. Adding further neurons was beneficial until five neurons, in the case of Raman and NIR-Raman input, or four neurons, in the case of NIR input. Error of models slightly increased after this threshold, implying that four or five neurons were enough to describe the behavior of this system, with more neurons only leading to overfitted models. It was also clear that Levenberg–Marquardt-based ANNs were worse in all cases when compared with Bayesian regularization models, although the latter took a longer time to train (with 10 neurons, 100 training steps took around 20 min for Bayesian regularization and 5 min for Levenberg–Marquardt); this difference in training time does not justify using Levenberg–Marquardt. The models with the lowest RMSEP value were chosen for further evaluation (five neuron models for Raman and NIR-Raman and four neuron models for NIR). The predictive ability of these models was compared to prediction yielded by a PLS model built using the same data and using the same input for validation. The predicted dissolution profile of these models was compared to the measured profiles by calculating the f_2_ value. Table 4 shows this comparison, with ANN models outperforming PLS models in all cases.

**Figure 8:**
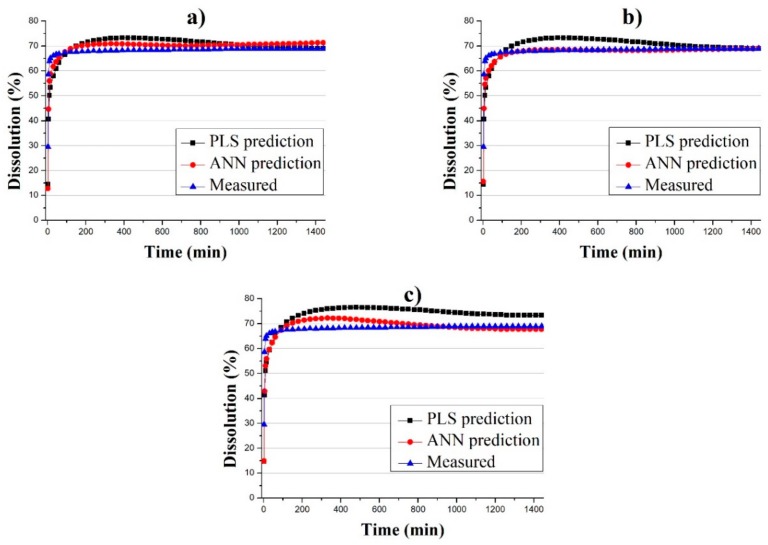
Average of predicted (PLS and ANN) and measured dissolution profiles of Formulation 1 tablets where predictions were based on (**a**) Raman, (**b**) NIR and (**c**) Raman and NIR spectra.

The main difference between the performance of ANN and PLS models was their ability to recognize the nonlinear effect of HPMC content on the dissolution curve. This difference can be observed in the case of 10% HPMC (Formulation 1) test tablets when the average of PLS and ANN predictions were plotted and compared with the measured profiles (Figure 8).

The average prediction of Formulation 1 tablets was more accurate when the ANN models were used. On the basis of these results, ANN is a better choice for predicting dissolution profiles as it gives better predictions, and yet constructing the model does not require more resources. Also, it is not necessary to combine the two spectroscopic methods, as predictions based on both individual NIR and Raman spectra yielded approximately the same result, with Raman being slightly better.

**Figure 9:**
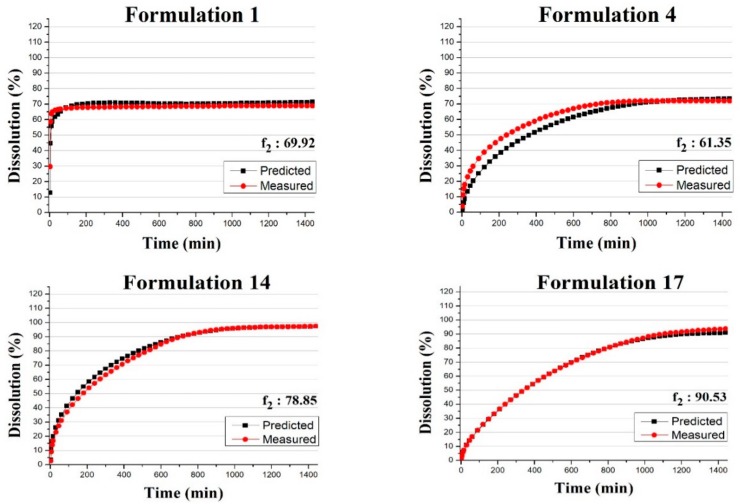

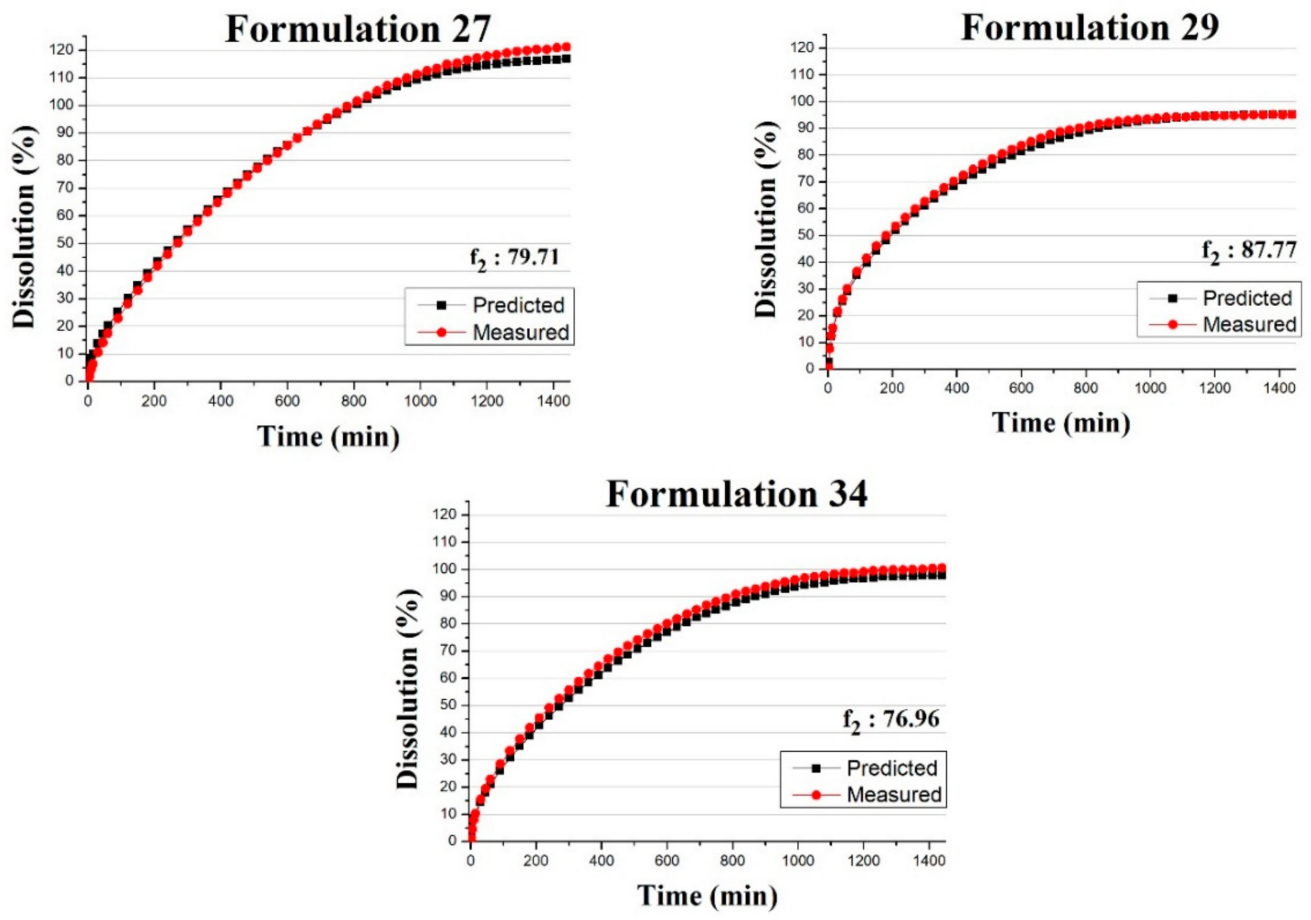
Average of predicted and measured dissolution profiles of validation tablets; inputs are based on Raman spectra.

The proposed method utilizing an ANN was capable of predicting the in vitro dissolution profile of all the test tablets within the acceptance limit of the f_2_ similarity factor (Figure 9). For the comparison of predicted and measured profiles of individual tablets and their f_2_ values, see Figure S7 and Table S1, respectively. This method used the three factors determining the shape of the dissolution profile as inputs of the ANN. These were the DR and HPMC content and the compression force. The first two were measured either by transmission Raman or NIR spectroscopy, and calculated from PLS models, while the compression force was measured by the tablet press. The advantage of this method was that while predicting the dissolution profile, it also yielded information on the composition of the tablets, which is a critical quality attribute. This could be exploited by reducing the number of analytical equipment used to monitor the tableting process, as fewer instruments can yield the same information. As this method used the real physical attributes of the tablets as input, it could be possible to measure these input values by other means without compromising the performance of the model. As a conclusion, the results of this work demonstrate that if the information collected during the production of tablets is processed in an optimal way, much more information can be gained on the quality of the product compared to current methods.

## 4. Conclusions

Recent advancements in spectroscopy and computer technology opened the way to a new era for the pharmaceutical industry, where in-line monitoring of the processes yields a tremendous amount of information. When processed with the right tools, this information enables a more comprehensive characterization of intermediates and end products. The current work aimed to utilize the data collected by NIR and Raman spectroscopy, along with the compression force measured by the tablet press. The DR and HPMC content and the compression force of the tablets were used as inputs in an ANN model, which aimed to predict the in vitro dissolution profile of the tablets. A total of 148 tablets were produced with 37 different settings of the aforementioned three variables. It was found that transmission Raman spectroscopy was best suited for the prediction of DR content, while in the case of HPMC content, transmission NIR spectroscopy gave the best results. ANNs were built based on two learning function methods, Levenberg–Marquardt and Bayesian regularization, with the latter yielding more accurate results. The predictions of ANN models were compared to predictions of PLS models using the same input. It was found that ANN is more capable of handling the nonlinearity of the effect of HPMC on the dissolution curve; thus, using ANN can be beneficial. Models can be built based on both NIR and Raman spectroscopy, which can predict the dissolution profile of the test tablets within the acceptance limit of the f_2_ similarity factor. These results imply that utilizing the proper data processing methods enables us to replace some of the most cumbersome analytical techniques with ones that require a minimal amount of human labor, yet can characterize a much larger fraction of the product—possible every single tablet. Further research could be carried out in order to construct models that consider the impact of more process parameters—possibly the particle size of the drug and key excipients, the degree of lubrication of the tablets or the quality of the film coating. These solutions, when adopted by the pharmaceutical industry, can lead to technologies where the quality of the product is understood to a much deeper extent, and thus it can be assured that the patient will receive a treatment of the desired quality.

## Figures and Tables

**Table 1:** Experimental conditions applied for the tablet manufacturing. Additional settings are displayed in italics. Settings chosen for validation are displayed in bold. DR = drotaverine; HPMC = hydroxypropyl methylcellulose.

Formulation Number	DR Content (*w*/*w* %)	HPMC Content (*w*/*w* %)	Compression Force (MPa)
**1**	**6**	**10**	**63.8**
2	8	10	63.8
3	10	10	63.8
**4**	**6**	**20**	**63.8**
5	8	20	63.8
6	10	20	63.8
7	6	30	63.8
8	8	30	63.8
9	10	30	63.8
10	6	10	95.7
11	8	10	95.7
12	10	10	95.7
13	6	20	95.7
**14**	**8**	**20**	**95.7**
15	10	20	95.7
16	6	30	95.7
**17**	**8**	**30**	**95.7**
18	10	30	95.7
19	6	10	127.6
20	8	10	127.6
21	10	10	127.6
22	6	20	127.6
23	8	20	127.6
24	10	20	127.6
25	6	30	127.6
26	8	30	127.6
**27**	**10**	**30**	**127.6**
*28*	*7*	*20*	*63.8*
***29***	***7.5***	***20***	***63.8***
*30*	*8.5*	*20*	*63.8*
*31*	*9*	*20*	*63.8*
*32*	*8*	*5*	*63.8*
*33*	*8*	*15*	*63.8*
***34***	***8***	***25***	***63.8***
*35*	*8*	*35*	*63.8*
*36*	*8*	*20*	*31.9*
*37*	*8*	*20*	*159.5*

**Table 2:** Parameters of the models predicting DR content (models gained after genetic algorithm (GA) runs are in parentheses).

Type of Data	Raman Transmission (GA)	Raman Reflection (GA)	NIR Transmission (GA)	NIR Reflection (GA)
Pretreatment method ^a^	bl, SNV, MC	SNV, MC	der, MSC, MC	der, MSC, MC
Spectral region (cm^−1^)	350–1680	350–1680	7600–8000, 8500–13,000	4200–7400
Number of LVs	2 (3)	4 (6)	3 (3)	6 (6)
R^2^_c_	0.911 (0.943)	0.893 (0.962)	0.905 (0.934)	0.750 (0.777)
R^2^_cv_	0.894 (0.934)	0.875 (0.928)	0.876 (0.912)	0.586 (0.700)
R^2^_p_	0.913 (0.905)	0.868 (0.778)	0.856 (0.918)	0.579 (0.444)
RMSEC (% *w*/*w*)	0.428 (0.343)	0.471 (0.281)	0.443 (0.370)	0.718 (0.680)
RMSECV (% *w*/*w*)	0.468 (0.370)	0.509 (0.386)	0.506 (0.426)	0.928 (0.789)
RMSEP (% *w*/*w*)	0.386 (0.400)	0.467 (0.602)	0.500 (0.414)	0.837 (0.977)

^a^ bl: baseline correction, SNV: standard normal variate, MC: mean centering, der: 1st derivative, MSC: multiplicative signal correction, LVs: latent variables, RMSEC: root mean square error of calibration, RMSECV: root mean square error of cross-validation, RMSEP: root mean square error of prediction.

**Table 3:** Parameters of the models predicting HPMC content (models gained after GA runs are in parentheses).

Type of Data	Raman Transmission (GA)	Raman Reflection (GA)	NIR Transmission (GA)	NIR Reflection (GA)
Pretreatment method ^a^	bl, SNV, MC	SNV, MC	der, MSC, MC	der, MSC, MC
Spectral region (cm^−1^)	350–1680	350–1680	7600–8000, 8500–13,000	4200–7400
Number of LVs	2 (5)	4 (4)	4 (4)	4 (4)
R^2^_c_	0.953 (0.986)	0.958 (0.966)	0.986 (0.988)	0.924 (0.951)
R^2^_cv_	0.947 (0.982)	0.950 (0.959)	0.983 (0.986)	0.907 (0.947)
R^2^_p_	0.956 (0.975)	0.956 (0.942)	0.982 (0.982)	0.875 (0.909)
RMSEC (% *w*/*w*)	1.753 (0.950)	1.654 (1.500)	0.949 (0.884)	2.231 (1.610)
RMSECV (% *w*/*w*)	1.862 (1.082)	1.811 (1.643)	1.049 (0.962)	2.470 (1.861)
RMSEP (% *w*/*w*)	1.381 (1.031)	1.443 (1.630)	0.861 (0.914)	2.307 (2.068)

^a^ bl: baseline correction, SNV: standard normal variate, MC: mean centering, der: 1st derivative, MSC: multiplicative signal correction.

**Table 4:** Average f_2_ value of the best artificial neural network (ANN) models compared to partial least squares (PLS) models using the same input.

Modeling Method	Raman	NIR	NIR-Raman
ANN	74.27	71.84	73.07
PLS	65.63	65.01	65.79

## Supplementary Materials

The following are available online at https://www.mdpi.com/1999-4923/11/8/400/s1, Figure S1: Loading plots of the constructed PCA models based on NIR transmission spectra: (**a**) PC1, (**b**) PC2, Figure S2: Loading plots of the constructed PCA models based on Raman transmission spectra: (**a**) PC1, (**b**) PC 2, Figure S3: PLS regression curve of model predicting DR content based on NIR transmission spectra. Grey circles are training samples, blue squares are test samples, Figure S4: PLS regression curve of model predicting HPMC content based on NIR transmission spectra. Grey circles are training samples, blue squares are test samples, Figure S5: PLS regression curve of model predicting DR content based on Raman transmission spectra. Grey circles are training samples, blue squares are test samples, Figure S6: PLS regression curve of model predicting HPMC content based on Raman transmission spectra. Grey circles are training samples, blue squares are test samples, Figure S7: Predicted and measured dissolution profile of all test tablets, Table S1: *f*_2_ values of the measured and predicted dissolution profile of test tablets.

## References

[1] Hédoux A. (2016). Recent developments in the Raman and infrared investigations of amorphous pharmaceuticals and protein formulations: A review. Adv. Drug Deliv. Rev..

[2] Porep J.U., Kammerer D.R., Carle R. (2015). On-line application of near infrared (NIR) spectroscopy in food production. Trends Food Sci. Technol..

[3] Esmonde-White K.A., Cuellar M., Uerpmann C., Lenain B., Lewis I.R. (2017). Raman spectroscopy as a process analytical technology for pharmaceutical manufacturing and bioprocessing. Anal. Bioanal. Chem..

[4] Lohumi S., Lee S., Lee H., Cho B.-K. (2015). A review of vibrational spectroscopic techniques for the detection of food authenticity and adulteration. Trends Food Sci. Technol..

[5] Paudel A., Raijada D., Rantanen J. (2015). Raman spectroscopy in pharmaceutical product design. Adv. Drug Deliv. Rev..

[6] Manley M., Baeten V. (2018). Spectroscopic Technique: Near Infrared (NIR) Spectroscopy. Modern Techniques for Food Authentication.

[7] Bumbrah G.S., Sharma R.M. (2016). Raman spectroscopy–Basic principle, instrumentation and selected applications for the characterization of drugs of abuse. Egypt. J. Forensic Sci..

[8] Das R.S., Agrawal Y. (2011). Raman spectroscopy: Recent advancements, techniques and applications. Vib. Spectrosc..

[9] Nagy B., Farkas A., Borbás E., Vass P., Nagy Z.K., Marosi G. (2019). Raman Spectroscopy for Process Analytical Technologies of Pharmaceutical Secondary Manufacturing. AAPS PharmSciTech.

[10] Arruabarrena J., Coello J., Maspoch S. (2014). Raman spectroscopy as a complementary tool to assess the content uniformity of dosage units in break-scored warfarin tablets. Int. J. Pharm..

[11] Nagy B., Farkas A., Gyürkés M., Komaromy-Hiller S., Démuth B., Szabó B., Nusser D., Borbás E., Marosi G., Nagy Z.K. (2017). In-line Raman spectroscopic monitoring and feedback control of a continuous twin-screw pharmaceutical powder blending and tableting process. Int. J. Pharm..

[12] Riolo D., Piazza A., Cottini C., Serafini M., Lutero E., Cuoghi E., Gasparini L., Botturi D., Marino I.G., Aliatis I. (2018). Raman spectroscopy as a PAT for pharmaceutical blending: Advantages and disadvantages. J. Pharm. Biomed. Anal..

[13] Harting J., Kleinebudde P. (2019). Optimisation of an in-line Raman spectroscopic method for continuous API quantification during twin-screw wet granulation and its application for process characterisation. Eur. J. Pharm. Biopharm..

[14] Nagy B., Farkas A., Magyar K., Démuth B., Nagy Z.K., Marosi G. (2018). Spectroscopic characterization of tablet properties in a continuous powder blending and tableting process. Eur. J. Pharm. Sci..

[15] Liu R., Li L., Yin W., Xu D., Zang H. (2017). Near-infrared spectroscopy monitoring and control of the fluidized bed granulation and coating processes—A review. Int. J. Pharm..

[16] Pauli V., Elbaz F., Kleinebudde P., Krumme M. (2018). Methodology for a variable rate control strategy development in continuous manufacturing applied to twin-screw wet-granulation and continuous fluid-bed drying. J. Pharm. Innov..

[17] Dégardin K., Guillemain A., Guerreiro N.V., Roggo Y. (2016). Near infrared spectroscopy for counterfeit detection using a large database of pharmaceutical tablets. J. Pharm. Biomed. Anal..

[18] Terra L.A., Poppi R.J. (2014). Monitoring the polymorphic transformation on the surface of carbamazepine tablets generated by heating using near-infrared chemical imaging and chemometric methodologies. Chemom. Intell. Lab. Syst..

[19] Pataki H., Csontos I., Nagy Z.K., Vajna B., Molnar M., Katona L., Marosi G. (2012). Implementation of Raman signal feedback to perform controlled crystallization of carvedilol. Org. Process Res. Dev..

[20] Pataki H., Markovits I., Vajna B.z., Nagy Z.K., Marosi G.R. (2012). In-line monitoring of carvedilol crystallization using Raman spectroscopy. Cryst. Growth Des..

[21] Sacré P.-Y., De Bleye C., Chavez P.-F., Netchacovitch L., Hubert P., Ziemons E. (2014). Data processing of vibrational chemical imaging for pharmaceutical applications. J. Pharm. Biomed. Anal..

[22] Vajna B., Patyi G., Nagy Z., Bódis A., Farkas A., Marosi G. (2011). Comparison of chemometric methods in the analysis of pharmaceuticals with hyperspectral Raman imaging. J. Raman Spectrosc..

[23] Farkas A., Vajna B., Sóti P.L., Nagy Z.K., Pataki H., Van der Gucht F., Marosi G. (2015). Comparison of multivariate linear regression methods in micro-Raman spectrometric quantitative characterization. J. Raman Spectrosc..

[24] Bro R., Smilde A.K. (2014). Principal component analysis. Anal. Methods.

[25] Wold H. (2004). Partial least squares. Encycl. Stat. Sci..

[26] Noyes A.A., Whitney W.R. (1897). The rate of solution of solid substances in their own solutions. J. Am. Chem. Soc..

[27] Dokoumetzidis A., Macheras P. (2006). A century of dissolution research: From Noyes and Whitney to the biopharmaceutics classification system. Int. J. Pharm..

[28] Ambrus R., Alshweiat A., Csóka I., Ovari G., Esmail A., Radacsi N. (2019). 3D-printed electrospinning setup for the preparation of loratadine nanofibers with enhanced physicochemical properties. Int. J. Pharm..

[29] Zupančič S., Sinha-Ray S., Sinha-Ray S., Kristl J., Yarin A.L. (2016). Controlled release of ciprofloxacin from core–Shell nanofibers with monolithic or blended core. Mol. Pharm..

[30] Krupa A., Tabor Z., Tarasiuk J., Strach B., Pociecha K., Wyska E., Wroński S., Łyszczarz E., Jachowicz R. (2018). The impact of polymers on 3D microstructure and controlled release of sildenafil citrate from hydrophilic matrices. Eur. J. Pharm. Sci..

[31] Lawrence X.Y. (2008). Pharmaceutical quality by design: Product and process development, understanding, and control. Pharm. Res..

[32] Pawar P., Wang Y., Keyvan G., Callegari G., Cuitino A., Muzzio F. (2016). Enabling real time release testing by NIR prediction of dissolution of tablets made by continuous direct compression (CDC). Int. J. Pharm..

[33] U.S. Department of Health and Human Services, Food and Drug Administration, Center for Drug Evaluation and Research (CDER) (2019). Quality Considerations for Continuous Manufacturing Guidance for Industry (Draft Guidence).

[34] Donoso M., Ghaly E.S. (2005). Prediction of drug dissolution from tablets using near-infrared diffuse reflectance spectroscopy as a nondestructive method. Pharm. Dev. Technol..

[35] Freitas M.P., Sabadin A., Silva L.M., Giannotti F.M., do Couto D.A., Tonhi E., Medeiros R.S., Coco G.L., Russo V.F., Martins J.A. (2005). Prediction of drug dissolution profiles from tablets using NIR diffuse reflectance spectroscopy: A rapid and nondestructive method. J. Pharm. Biomed. Anal..

[36] Hernandez E., Pawar P., Keyvan G., Wang Y., Velez N., Callegari G., Cuitino A., Michniak-Kohn B., Muzzio F.J., Romañach R.J. (2016). Prediction of dissolution profiles by non-destructive near infrared spectroscopy in tablets subjected to different levels of strain. J. Pharm. Biomed. Anal..

[37] Sutariya V., Groshev A., Sadana P., Bhatia D., Pathak Y. (2013). Artificial neural network in drug delivery and pharmaceutical research. Open Bioinform. J..

[38] Yegnanarayana B. (2009). Artificial Neural Networks.

[39] Potdar A., Longstaff A.P., Fletcher S., Mian N.S. (2015). Application of Multi Sensor Data Fusion Based on Principal Component Analysis and Artificial Neural Network for Machine Tool Thermal Monitoring.

[40] Edwards C. (2015). Growing pains for deep learning. Commun. ACM.

[41] Ibrić S., Djuriš J., Parojčić J., Djurić Z. (2012). Artificial neural networks in evaluation and optimization of modified release solid dosage forms. Pharmaceutics.

[42] Ilić M., Đuriš J., Kovačević I., Ibrić S., Parojčić J. (2014). In vitro–In silico–In vivo drug absorption model development based on mechanistic gastrointestinal simulation and artificial neural networks: Nifedipine osmotic release tablets case study. Eur. J. Pharm. Sci..

[43] Montañez-Godínez N., Martínez-Olguín A.C., Deeb O., Garduño-Juárez R., Ramírez-Galicia G. (2015). QSAR/QSPR as an Application of Artificial Neural Networks. Artificial Neural Networks.

[44] Moss G.P., Sun Y., Wilkinson S.C., Davey N., Adams R., Martin G.P., Prapopopolou M., Brown M.B. (2011). The application and limitations of mathematical modelling in the prediction of permeability across mammalian skin and polydimethylsiloxane membranes. J. Pharm. Pharmacol..

[45] Lou H., Chung J.I., Kiang Y.-H., Xiao L.-Y., Hageman M.J. (2019). The application of machine learning algorithms in understanding the effect of core/shell technique on improving powder compactability. Int. J. Pharm..

[46] Millen N., Kovačević A., Djuriš J., Ibrić S. (2019). Machine Learning Modeling of Wet Granulation Scale-up Using Particle Size Distribution Characterization Parameters. J. Pharm. Innov..

[47] Han R., Yang Y., Li X., Ouyang D. (2018). Predicting oral disintegrating tablet formulations by neural network techniques. Asian J. Pharm. Sci..

[48] Ebube N.K., McCall T., Chen Y., Meyer M.C. (1997). Relating formulation variables to in vitro dissolution using an artificial neural network. Pharm. Dev. Technol..

[49] Peh K.K., Lim C.P., San Quek S., Khoh K.H. (2000). Use of artificial neural networks to predict drug dissolution profiles and evaluation of network performance using similarity factor. Pharm. Res..

[50] Helland I. (2014). Partial Least Squares Regression. Wiley StatsRef: Statistics Reference Online.

[51] Moore J.W. (1996). Mathematical comparison of dissolution profiles. Pharm. Technol..

[52] O’hara T., Dunne A., Butler J., Devane J., Group I.C.W. (1998). A review of methods used to compare dissolution profile data. Pharm. Sci. Technol. Today.

[53] Démuth B., Nagy Z.K., Balogh A., Vigh T., Marosi G., Verreck G., Van Assche I., Brewster M. (2015). Downstream processing of polymer-based amorphous solid dispersions to generate tablet formulations. Int. J. Pharm..

