# Supplementary material for: Fast, Spectroscopy-Based Prediction of In Vitro Dissolution Profile of Extended Release Tablets Using Artificial Neural Networks
Source: Pharmaceutics. 2019 Aug 9;11(8):400. doi: 10.3390/pharmaceutics11080400 (PMC6723897; doi:10.3390/pharmaceutics11080400)
Supplement: Supplementary file 1 [file pharmaceutics-11-00400-s001.pdf]

pharmaceutics

MDPI

Supplementary Materials: Fast, Spectroscopy-Based Prediction of In Vitro Dissolution Profile of Extended Release Tablets Using Artificial Neural Networks

Dorián László Galata, Attila Farkas, Zsófia Könyves, Lilla Alexandra Mészáros, Edina Szabó, István Csontos, Andrea Pálos, György Marosi, Zsombor Kristóf Nagy and Brigitta Nagy

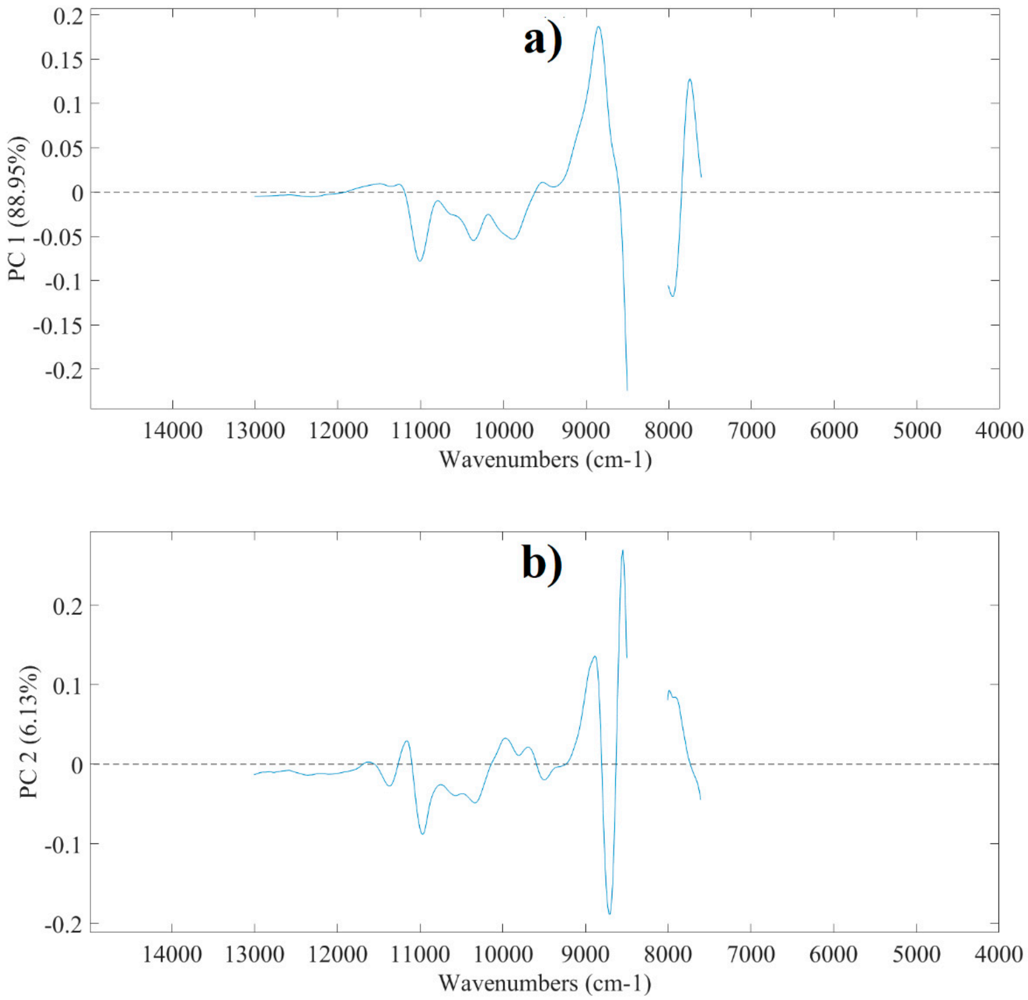

Figure S1. Loading plots of the constructed PCA models based on NIR transmission spectra: (**a**) PC1, (**b**) PC2.

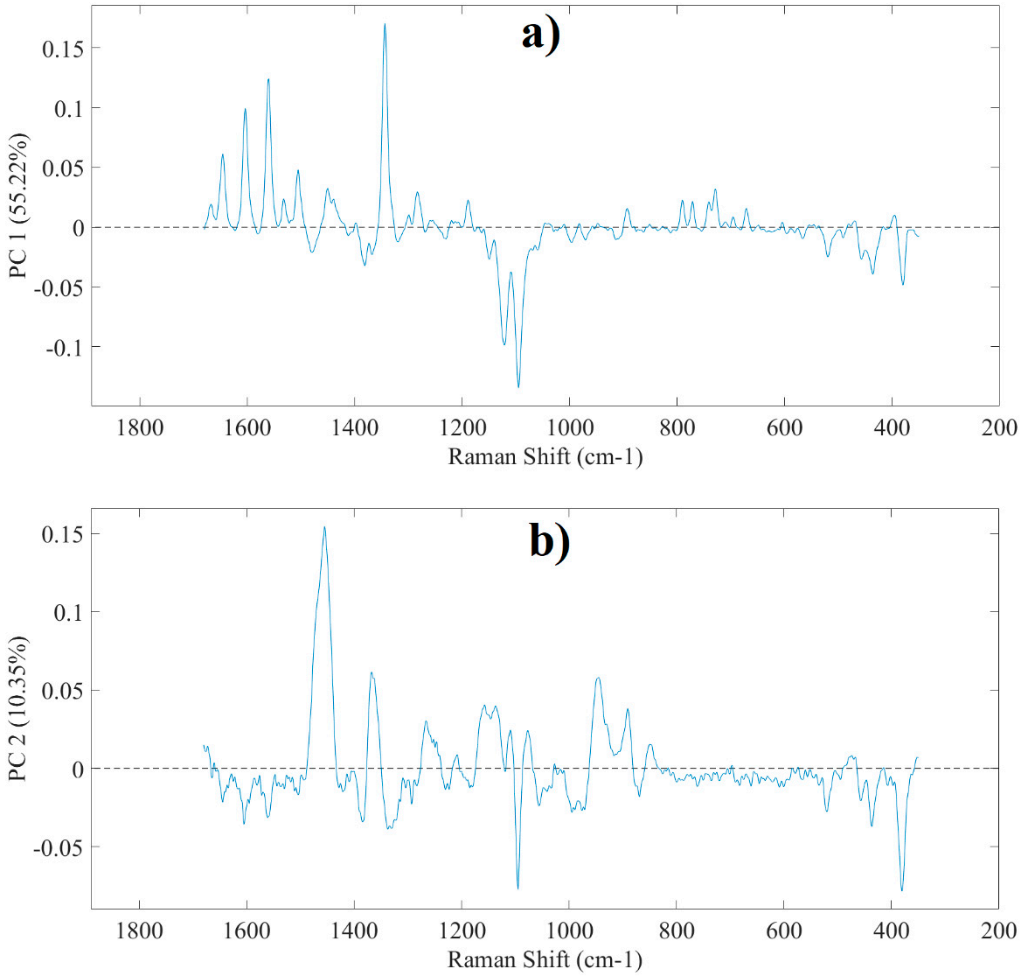

Figure S2. Loading plots of the constructed PCA models based on Raman transmission spectra: (**a**) PC1, (**b**) PC 2.

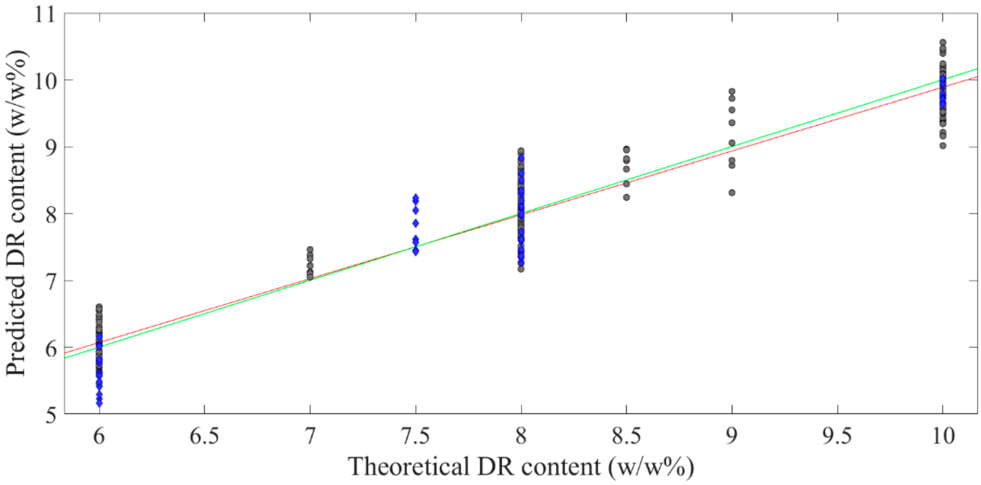

Figure S3. PLS regression curve of model predicting DR content based on NIR transmission spectra. Grey circles are training samples, blue squares are test samples.

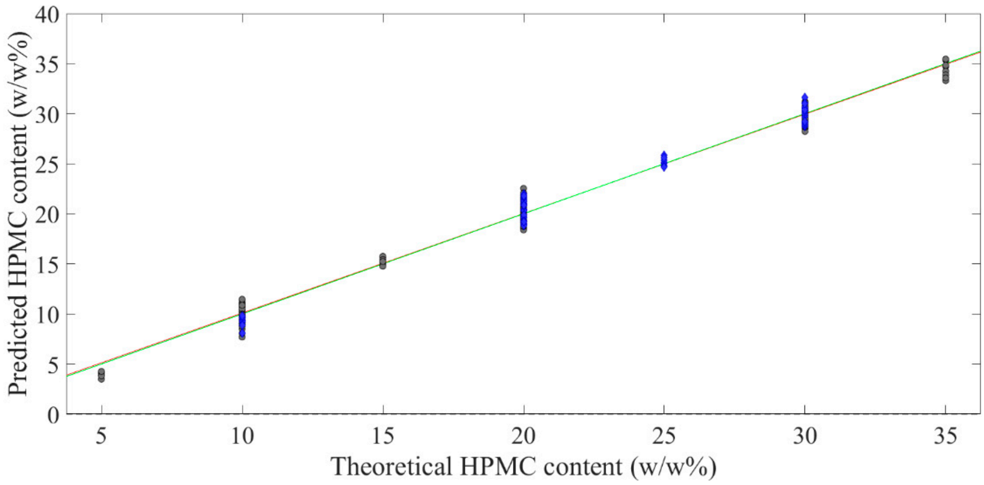

Figure S4. PLS regression curve of model predicting HPMC content based on NIR transmission spectra. Grey circles are training samples, blue squares are test samples.

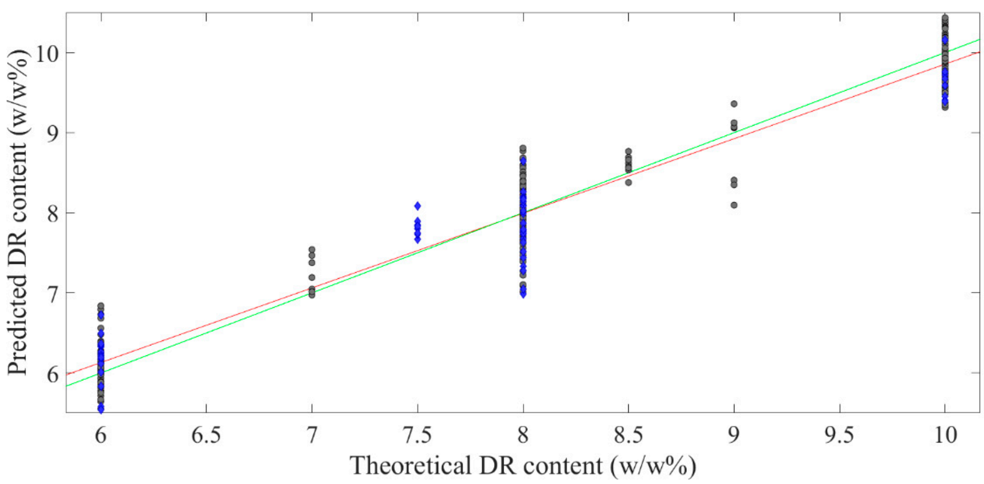

Figure S5. PLS regression curve of model predicting DR content based on Raman transmission spectra. Grey circles are training samples, blue squares are test samples.

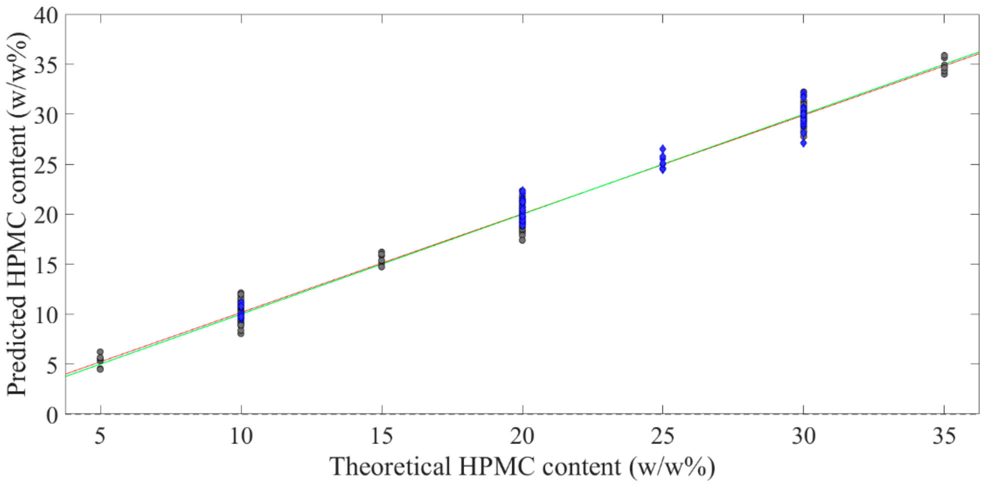

Figure S6. PLS regression curve of model predicting HPMC content based on Raman transmission spectra. Grey circles are training samples, blue squares are test samples.

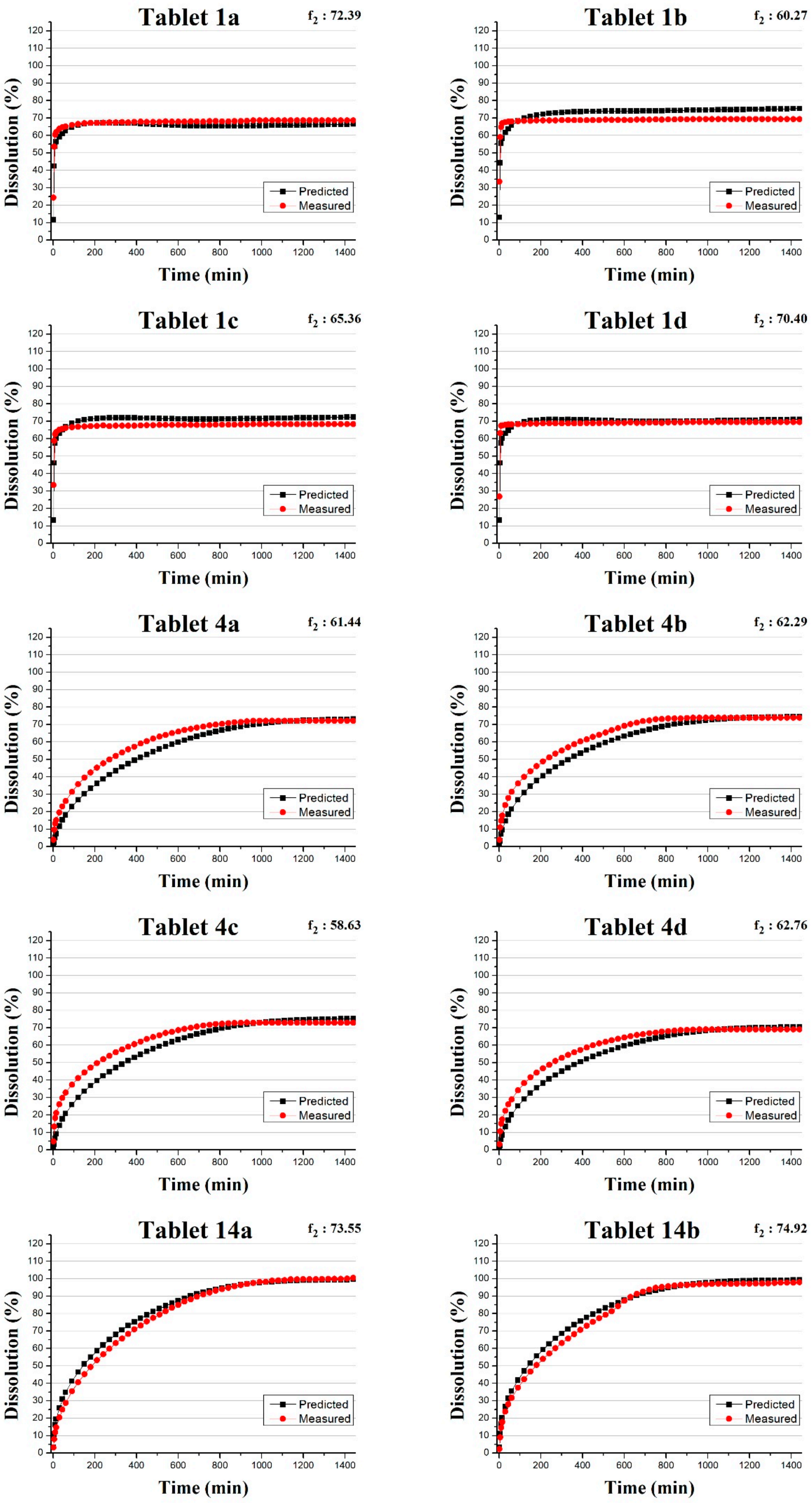
Tablet 1a
f2 : 72.39
Tablet 1b
f2 : 60.27
Tablet 1c
f2 : 65.36
Tablet 1d
f2 : 70.40
Tablet 4a
f2 : 61.44
Tablet 4b
f2 : 62.29
Tablet 4c
f2 : 58.63
Tablet 4d
f2 : 62.76
Tablet 14a
f2 : 73.55
Tablet 14b
f2 : 74.92
Dissolution (%)
Time (min)
Predicted
Measured

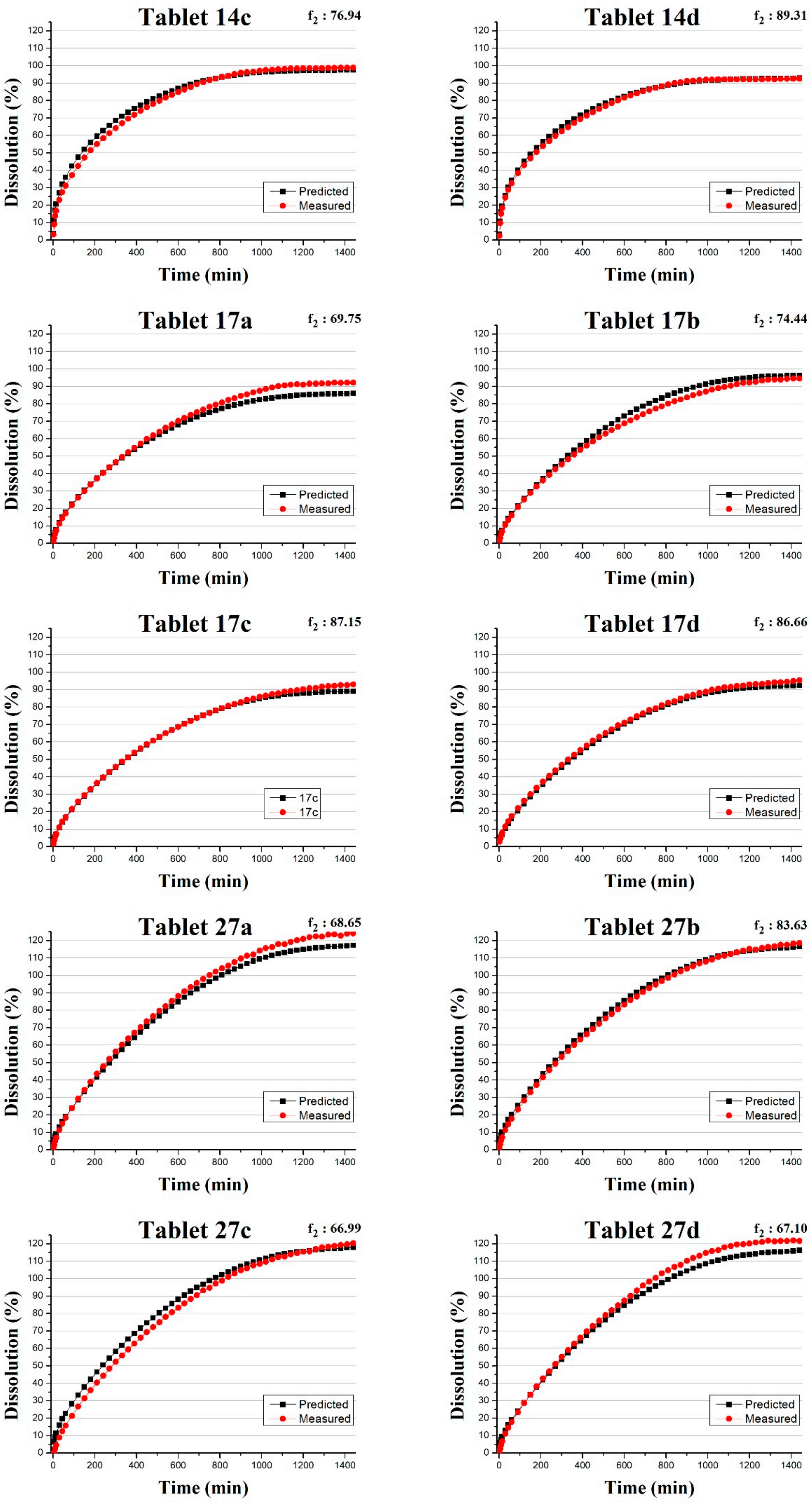

Tablet 14c
f2 : 76.94
Tablet 14d
f2 : 89.31
Tablet 17a
f2 : 69.75
Tablet 17b
f2 : 74.44
Tablet 17c
f2 : 87.15
Tablet 17d
f2 : 86.66
Tablet 27a
f2 : 68.65
Tablet 27b
f2 : 83.63
Tablet 27c
f2 : 66.99
Tablet 27d
f2 : 67.10
Dissolution (%)
Time (min)
Predicted
Measured
17c

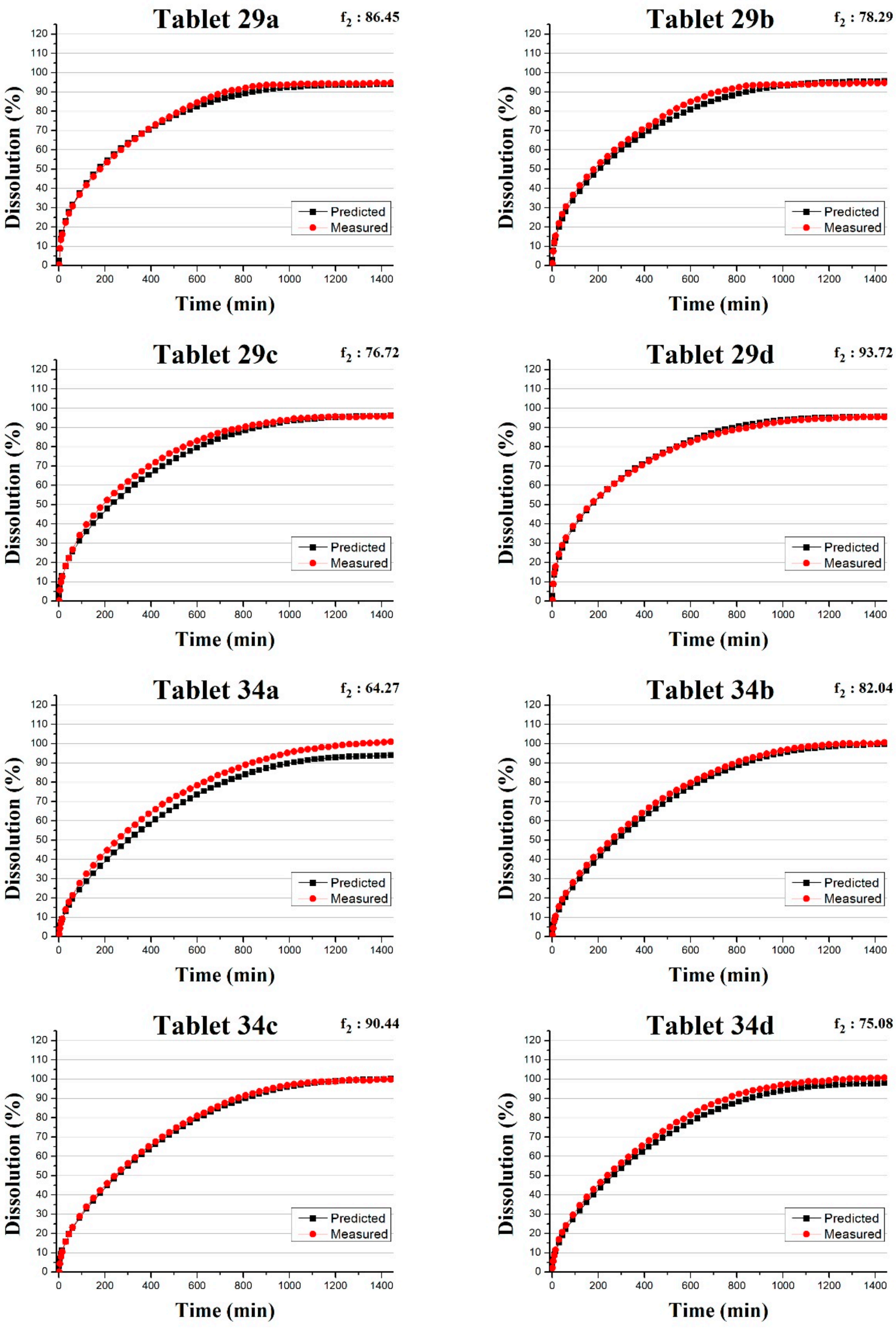

Figure S7. Predicted and measured dissolution profile of all test tablets.

Table S1. f_2 values of the measured and predicted dissolution profile of test tablets.

Tablet Name	f_2 Value	Tablet Name	f_2 Value	Tablet Name	f_2 Value
1a	72.39	17a	69.75	34a	64.27
1b	60.27	17b	74.44	34b	82.04
1c	65.36	17c	87.15	34c	90.44
1d	70.40	17d	86.66	34d	75.08
4a	61.44	27a	68.65		
4b	62.29	27b	83.63		
4c	58.63	27c	66.99		
4d	62.76	27d	67.10		
14a	73.55	29a	86.45		
14b	74.92	29b	78.29		
14c	76.94	29c	76.72		
14d	89.31	29d	93.72		
